# Development and Implementation of an HPV Vaccination Survey for American Indians in Cherokee Nation

**DOI:** 10.3390/ijerph18179239

**Published:** 2021-09-01

**Authors:** Sameer Vali Gopalani, Amanda E. Janitz, Margie Burkhart, Janis E. Campbell, Sydney A. Martinez, Ashley H. White, Sixia Chen, Amber S. Anderson, Stephanie F. Pharr, Scott Patrick, Ashley Comiford

**Affiliations:** 1 Hudson College of Public Health, University of Oklahoma Health Sciences Center, Oklahoma City, OK 73104, USA; Amanda-Janitz@ouhsc.edu (A.E.J.); Janis-Campbell@ouhsc.edu (J.E.C.); Sydney-Martinez@ouhsc.edu (S.A.M.); ashley-white@ouhsc.edu (A.H.W.); Sixia-Chen@ouhsc.edu (S.C.); Amber-S-Anderson@ouhsc.edu (A.S.A.); 2 Cherokee Nation Public Health, Tahlequah, OK 74464, USA; Margie-Burkhart@cherokee.org (M.B.); scottpatrick24@gmail.com (S.P.); Ashley-Comiford@cherokee.org (A.C.); 3 OU Health Stephenson Cancer Center, Oklahoma City, OK 73104, USA; Stephanie-Pharr@ouhsc.edu

**Keywords:** American Indian, HPV vaccination, indigenous data sovereignty, survey design, questionnaire

## Abstract

Improving human papillomavirus (HPV) vaccination rates is a public health priority and a crucial cancer prevention goal. We designed a survey to estimate HPV vaccination coverage and understand factors associated with HPV vaccination among American Indian adolescents aged 9 to 17 years in Cherokee Nation, United States. The final survey contains 37 questions across 10 content areas, including HPV vaccination awareness, initiation, reasons, recommendations, and beliefs. This process paper provides an overview of the survey development. We focus on the collaborative process of a tribal–academic partnership and discuss methodological decisions regarding survey sampling, measures, testing, and administration.

## 1. Introduction

American Indian and Alaska Native women bear a disproportionate burden of cervical and other human papillomavirus (HPV)-associated cancers nationally and in Oklahoma [1,2]. From 2013 to 2017, the incidence of HPV-associated cancers nationally was 1.2 times higher in American Indian and Alaska Native (15.9 per 100,000) than non-Hispanic White (13.7 per 100,000) women [1]. In addition, during the same period, regional variations in HPV-associated cancers among American Indian and Alaska Native populations have been observed, with the highest incidence of all HPV-associated cancers among males (14.9 per 100,000) and cervical cancer among females (13.8 per 100,000) reported in the Southern Plains (Kansas, Oklahoma, and Texas) [1]. In Oklahoma, American Indian and Alaska Native women had the highest age-adjusted incidence rate for cervical cancer (14.8 per 100,000 women) among all racial and ethnic groups. This incidence rate was significantly higher than the rate in White (rate ratio [RR]: 1.6; 95% confidence interval [CI]: 1.5, 1.8) and African American (RR: 1.5; 95% CI: 1.3, 1.8) women [2].

Vaccines to prevent HPV-associated cancers have been available and recommended for use in the United States (US) since their introduction in 2006 [3]. However, data on HPV vaccination coverage for individual American Indian and Alaska Native tribes are not readily available. In addition, patterns and policies for vaccination among American Indian and Alaska Native populations differ. For example, in a survey of HPV vaccination policies among American Indian tribes in Washington State, 25% of the facilities serving tribal members had a clinic-only policy, 4% had a tribal-only policy, 18% had both clinic and tribal policies, and 46% had no policy [4]. While tribal policies regarding HPV vaccine administration followed guidelines from the Advisory Committee on Immunization Practices (ACIP), some clinics followed guidelines from the ACIP, Vaccines for Children (VFC) program, or Washington State [4]. Examples of differing clinic policies included providing the HPV vaccine unless the family opted out, developing a standing order protocol to enable nurses to administer the vaccine without a physician’s order, and offering vaccines to 19-to-26-year-olds who may not meet the VFC eligibility criteria [4]. Similar to differences in HPV-associated cancer burden, these variations in policies likely result in regional- and tribal-dependent differences in HPV vaccination coverage. Furthermore, the lack of tribal-specific or missing estimates for HPV vaccination likely hinders the opportunity to monitor trends and improve coverage in their tribal communities.

Compared with other racial and ethnic groups in the US, surveys assessing HPV vaccination coverage and factors among American Indian and Alaska Native people are limited. Among the surveys focused on American Indian and Alaska Native populations, most examined HPV vaccination policies and practices among health care professionals [4,5,6,7]; some examined HPV vaccination decision-making in American Indian college students [8,9,10]; and few examined attitudes on HPV vaccination among American Indian women [11] and Alaska Native parents [12]. Other survey studies including American Indian and Alaska Native communities have investigated HPV vaccine-related knowledge [13] and HPV vaccine information sources [14]. As HPV vaccination coverage and factors differ by tribe, and in the absence of any study assessing these factors in Cherokee Nation, we developed a survey to address this research gap.

This article is part of a larger ongoing project on HPV vaccination in Cherokee Nation, which also includes assessing perceptions and attitudes towards HPV vaccination among parents through focus groups. Cherokee Nation Public Health initiated these HPV vaccination quantitative and qualitative analyses projects to inform their public health programs better. The focus of this article is to provide an overview of the design and implementation of an American Indian HPV vaccination survey currently underway in Cherokee Nation. In this process paper, we describe the community partnership, instrument development, testing, sampling, and survey dissemination. We also present, wherever applicable, checklist items from the reporting guidelines of the CONSIDER statement (Consolidated criteria for strengthening reporting of health research involving indigenous peoples) [15].

## 2. Methods

### 2.1. Research Team

The research team for this survey project consisted of the Cherokee Nation Comprehensive Cancer (CNCC) program director (MB), Cherokee Nation epidemiologist (AC), Cherokee Nation Public Health employees (SP; MF and KNP), and researchers from the University of Oklahoma Health Sciences Center (OUHSC) who are members of a federally recognized tribe (AJ, SAM, ASA) or who each have experience working with American Indian tribes in Oklahoma (SVG, JEC, AHW, SC, and SFP) (CONSIDER statement #6 and #7). The research team formed a working group in May 2019 and has had bi-weekly project meetings since June 2019. The process, as described below, was iterative with the ultimate goal of improving the health of Cherokee Nation citizens while respecting tribal sovereignty. Further, the epidemiologist (AC) is an early stage investigator at Cherokee Nation interested in reducing health disparities among American Indians, especially among Cherokee citizens. This project aims to facilitate her goal of research independence and increase the evaluation and research capacity at Cherokee Nation that will lead to tribally driven and community-engaged evaluation and research (CONSIDER statement #13). In addition, the process is part of the practice of data decolonization of evaluation and research in indigenous communities [16].

### 2.2. Survey Aims

Before the survey was developed, the aims of the HPV vaccination in Cherokee Nation study were discussed and refined. The overall study aims to: (1) estimate HPV vaccine initiation, defined as the receipt of at least one dose of the HPV vaccine, among American Indian adolescents aged 9 to 17 years accessing Cherokee Nation Health Services in Oklahoma, and (2) identify factors associated with HPV vaccine acceptance or refusal by parents or guardians of American Indian adolescents aged 9 to 17 years accessing Cherokee Nation Health Services. This study also aims to collect data on HPV vaccine awareness, completion, intentions, and beliefs.

After finalizing the aims, the best approach to achieve the study aims was carefully considered, and following discussions, the research team concluded that a survey would be the most appropriate tool.

### 2.3. Population

Located in northeastern Oklahoma, Cherokee Nation is the largest federally recognized tribe in the US, with more than 389,000 citizens. Approximately 37% of the tribal population lives on the reservation, spanning 14 counties and more than 7000 square miles in Oklahoma [17]. The Cherokee Nation Health Services serve over 100,000 patients through two inpatient and nine outpatient health facilities throughout the reservation [18].

As parents and guardians play a prominent role in the vaccine uptake and vaccination behaviors of their children [19], the target population for this survey was parents or guardians of American Indian adolescents aged 9 to 17 years who used the Cherokee Nation Health Services from 1 January 2018 to 31 August 2020.

### 2.4. Sampling and Sample Size

We used electronic health records (EHR) from Cherokee Nation Health Services as the sampling frame. Using EHR as the frame reduced the potential for coverage error—the bias that occurs when the sampling frame does not cover the target population [20]. From 1 January 2018 to 31 August 2020, 15,803 adolescents aged 9 to 17 years were seen at the Cherokee Nation Health Services. Using this frame, a sample size of 2000 was drawn through a simple random sampling without replacement design in SAS (version 9.4; SAS Institute, Cary, NC, USA) [21].

For this survey, the sampling and observational units for this survey were individual parents or guardians of American Indian adolescents aged 9 to 17 years. Based on the HPV vaccination coverage of 70.1% for at least one dose among American Indian and Alaska Native teens nationally [22], the sample size needed for the survey was estimated to be 257, with a ±4% margin of sampling error.

### 2.5. Survey Measures

We selected survey measures based on how well the measures addressed the research aims and aligned with the priorities of Cherokee Nation Public Health and Cherokee Nation policymakers. To inform the selection and development of survey measures, the research team reviewed the Cherokee Nation Comprehensive Cancer Prevention and Control Plan 2018–2022 [17] and undertook a literature review of existing instruments on HPV vaccination.

Based on the literature review, we identified and examined adult- and adolescent-directed surveys with questions on HPV vaccination, including the National Immunization Survey (NIS)–Teen (2019) [23], National Health Interview Survey (NHIS) (2019) [24], Health Information National Trends Survey (HINTS) (2018) [25], National Survey of Family Growth (NSFG) (2015–2017) [26], National Health and Nutrition Examination Survey (NHANES) (2017–2018) [27], Behavioral Risk Factor Surveillance System (BRFSS) (2019) [28], UNC National Parents Survey (2017) [29], and Carolina HPV Immunization Attitudes and Beliefs Scale (CHIAS) (2007) [30]. In addition, we searched Q-Bank, which provides access to tested questions from over 50 national surveys [31]. These standardized questionnaires have been extensively tested and shown to have valid and reliable items, thereby reducing the potential of measurement error stemming from the instrument. However, before inclusion, we reviewed the question text, response options, reference period, respondents, data collection method, survey coverage, sample description, and any additional information about the question or scale. As a result, we adapted some of these questions for our survey.

#### 2.5.1. HPV Vaccine Awareness

Awareness of the HPV vaccine is a strong predictor of vaccine initiation [32,33]. In this survey, we assessed HPV vaccine awareness by asking, “A vaccine to prevent HPV infection is available. The vaccine is also known as or sometimes called the HPV vaccine, cervical cancer vaccine, Gardasil, or Cervarix. Before this survey, had you ever heard of the HPV vaccine?” Response options for the question included “yes” and “no”.

#### 2.5.2. HPV Vaccine Initiation

One of the main study outcomes, HPV vaccine initiation, was defined as the reported receipt of at least one dose of the vaccine. We estimated initiation through the question: “Has your child ever received an HPV shot or vaccine?” Response options were “yes”, “no”, and “I don’t know”. Estimating HPV vaccine initiation among American Indian adolescents accessing Cherokee Nation Health Services aligns with the strategy outlined in objective 1.3 of the Cherokee Nation Comprehensive Cancer Prevention and Control Plan 2018–2022 to increase HPV vaccination rates [17] (CONSIDER statement #4).

#### 2.5.3. HPV Vaccination Factors

To understand HPV vaccination factors, another primary outcome of interest, we ascertained the main reason for HPV vaccination refusal and acceptance. We assessed HPV vaccination refusal through the following question: “If your child has NOT received any shots of the HPV vaccine, what is the MAIN reason that your child has NOT received the HPV shot or vaccine? (select ONE response only).” Similarly, we assessed HPV vaccination receipt by asking: “If your child has received any shots of the HPV vaccine, what is the MAIN reason that your child DID receive the HPV shot or vaccine? (select ONE response only)”.

#### 2.5.4. HPV Vaccine Recommendation

As provider recommendation is a strong predictor of HPV vaccine initiation [34,35,36], we assessed this measure in our survey by asking the following question: “Has a doctor, nurse, or other health care provider ever recommended that your child receive an HPV shot or vaccine?” If the HPV vaccine was recommended by a provider, we also asked, “At what age did the doctor, nurse, or other health care provider recommend that your child should start receiving the HPV shot or vaccine?”

#### 2.5.5. Adolescent Vaccinations

To compare HPV vaccination coverage with other recommended vaccines for adolescents, we included questions about meningitis and tetanus booster shots in the survey. The question on meningitis vaccination read as: “Has your child ever received a meningitis shot or vaccine, sometimes called Menactra, Menveo, or Menomune?” The question on tetanus booster vaccination read as: “Has your child ever received a tetanus booster shot? This is usually given at 11–12 years of age. The tetanus booster shot is also known as or sometimes called the Td or Tdap shot or vaccine.” Response options for both questions were “yes”, “no”, and “I don’t know”.

#### 2.5.6. HPV Vaccine Beliefs

Participants were presented with statements on HPV vaccine importance, intent, and safety and were asked to rate agreement or disagreement using a five-point Likert scale of “strongly disagree”, “somewhat disagree”, “neither disagree or agree”, “somewhat agree”, and “strongly agree”. For instance, we assessed beliefs on the vaccine’s safety by these two statements: “the HPV vaccine can cause side effects, such as fever and discomfort” and “the HPV vaccine can cause lasting health problems”.

#### 2.5.7. Sociodemographic Variables

We ascertained sociodemographic information about adolescents and their parents or guardians. For American Indian adolescents, we asked questions about their gender, race, ethnicity, and age. For parents or guardians of American Indian adolescents, we asked questions about their gender, race, ethnicity, age, relationship to the adolescent, marital status, and highest level of education.

#### 2.5.8. COVID-19 and Vaccination

We also assessed COVID-19 vaccination intent among parents and adolescents by asking the following two questions: “How likely are you to get an approved COVID-19 vaccine when it becomes available?” and “How likely are you to get an approved COVID-19 vaccine for your child when it becomes available?” To ascertain whether the pandemic has caused any difficulty in getting the HPV vaccine, we also asked, “Has the COVID-19 pandemic made it difficult to get the HPV vaccine for your child?” These questions were selected and included in our survey as part of the Rapid Acceleration of Diagnostics-Underserved Populations (RADx^SM^-UP) Native American Research Centers for Health (NARCH) program for Cherokee Nation (Grant Number: 3S06GM127983-03S1).

### 2.6. Questionnaire Check

To ensure clarity and reduce the potential for measurement error, we avoided questions that were leading, loaded, double-barreled, negatively worded, and vague. We also avoided any acronyms or abbreviations. The question on HPV vaccination intent was asked twice in different sections of the survey to check for consistency of participant responses. We also assessed the readability of the survey using the Flesch Reading Ease score. We obtained a Flesch Reading Ease score of 64.6 out of 100 using Microsoft Word (version 2016; Microsoft Corporation, Redmond, WA, USA) and a score of 79.5 using an online Readability Test Tool (WebFX, Harrisburg, PA, USA) [37], indicating that the reading difficulty ranged from standard to fairly easy [38].

### 2.7. Survey Testing

Expert- and respondent-driven testing of the survey questions was undertaken in multiple ways, including stakeholder meetings, testing, and cognitive interviews.

#### 2.7.1. AIDCoP Meeting

At the American Indian Data Community of Practice (AIDCoP) meeting on 16 October 2019, in Oklahoma City, we tested questions on the primary outcomes and HPV vaccine beliefs. The organization’s membership includes over 80 members from 12 tribal nations and 10 sectors of community, state, tribal, and federal entities. Before the meeting, we shared the background and objectives of the survey with meeting participants.

For each question on the primary outcomes and HPV vaccine beliefs, AIDCoP members were asked to provide feedback by discussing and responding to: “What is the best way to ask this question? Are the response options appropriate? Do any response options need to be modified? Are there any response options that are missing (that should have been included)?” Discussion points and suggestions to improve the question and response options were captured and incorporated to update the survey.

#### 2.7.2. Cognitive Interviews

As part of the testing process, we conducted cognitive interviews to ensure that the respondents understood the survey questions. The interview was designed to elucidate comprehension of the survey question and understanding of the response process.

Seven participants were enrolled for cognitive interviews, as this sample size was considered adequate for identifying the measurement errors that the interviews were designed to detect [39]. We recruited participants through an e-mail message sent out to all Cherokee Nation employees.

We employed the think-aloud method (e.g., “tell me what you are thinking”) and asked the participants to actively verbalize their thoughts as they answered survey items [20,40]. We used the think-aloud method to identify any problems in answering the survey questions [20]. We also used the verbal probing method (e.g., “what does the term vaccination intent mean to you?”) to elicit detailed information beyond that usually provided by respondents [20,40]. The verbal probing method allowed us to assess participants’ understanding of the question and its key terms [20]. The interviews took place in September 2020 over Zoom (Zoom Video Communications, Inc., San Jose, CA, USA).

### 2.8. Survey Mode and Administration

The research team was unable to identify the parents or guardians by name specifically. Therefore, the team mailed the surveys to the listed address of the randomly selected adolescents and addressed the survey cover letter to “the parents/guardians of <INSERT ADOLESCENT’S NAME>”. Parents (biological, step, foster, adoptive) or guardians (grandparent, elder sibling, aunt or uncle, or other) of American Indian adolescents received paper surveys through the mail in April 2021. Either parent could complete the survey, or both parents could complete the survey together. The paper survey contained a weblink to an online version of the survey, offering participants the opportunity to take the survey either on paper or online through REDCap (Research Electronic Data Capture) (version 10.0.1; Vanderbilt University, Nashville, TN, USA). REDCap was selected as it is a secure, HIPAA-compliant web-based application designed for data collection for surveys. Before moving the REDCap survey to production, we thoroughly tested the survey for navigational and compatibility issues using various internet browsers on different platforms and operating systems. The survey will be self-administered. REDCap access to confidential information was limited to Cherokee Nation researchers and staff, and paper surveys were returned directly to Cherokee Nation. Only de-identified data were shared with researchers from the University of Oklahoma Health Sciences Center for analysis (CONSIDER statement #8).

In mixed-mode surveys, inconsistencies in presentation may lead to inconsistencies in measurement [41]. To reduce the potential for this measurement error, we adhered to the guidelines with the underlying principle of universal presentation developed by the U.S. Census Bureau [42]. The principle of universal presentation states that all respondents should be presented with the same question and response categories, regardless of mode, i.e., the meaning and intent of the question and response options must be consistent [42]. One of the trade-offs of the universal presentation was to eliminate skip logic from the online version of the survey as it could have created confusion in the paper survey, as seen in other studies [43].

To inform the prospective participants about the survey, an advance letter from Cherokee Nation Public Health was sent before the survey. As the letter came from a recognized organization in the community, participants may be more likely to respond to the survey, potentially reducing nonresponse error [44]. Furthermore, a round of reminder letters with the survey was sent to a subset of non-respondents in June 2021 to improve response and reduce nonresponse error. The survey was initially disseminated in April 2021, and we collected participant responses until July 2021. Data analysis will commence in August 2021.

### 2.9. Ethics

The study was approved by the Institutional Review Boards (IRB) of Cherokee Nation (approved on 27 July 2020) and the University of Oklahoma Health Sciences Center (IRB Number 12246; approved on 30 July 2020) and was conducted in compliance with its requirements (CONSIDER statement #5). To compensate for participation and reduce nonresponse error [45,46], we provided participants with a USD 30 gift card for the cognitive interview and a USD 10 gift card for the survey. The amount was agreed upon to be enough for appropriately compensating participants for their time and valuable contribution.

For cognitive interviews, we obtained verbal consent from participants using an information sheet. For the survey, we obtained a waiver of signed informed consent and added the following statement at the end of the consent form: “by taking the survey, you are agreeing to participate in this study” (CONSIDER statement #10).

The Cherokee Nation IRB reviewed and approved this paper before submission to the journal (CONSIDER statement #16).

## 3. Results

Based on the feedback from the AIDCoP meeting and cognitive interviews, the survey underwent several revisions. Participants provided input on survey measures, content, structure, terminology, and formatting (Table 1). For instance, we added a new question on assessing the awareness of HPV vaccine recommendation based on the cognitive interviews: “Before this survey, did you know that an HPV shot or vaccine was recommended for children aged 9–17 years?”

Participants also provided feedback on terminology, such as using “health care provider” instead of “healthcare professional” or “health care worker” in the survey questions; consistency, such as using HPV vaccine or shot throughout the survey instead of just HPV vaccine; formatting, such as bolding and capitalizing words in the question for the main measure, “If your child has **NOT** received any shots of the HPV vaccine, what is the **MAIN** reason that your child has **NOT** received the HPV shot or vaccine? (select **ONE** response only)”; and simplifying words, such as “risk” instead of “probability” and “getting” instead of “acquiring” in the response option “my child has a high risk of getting an HPV infection”.

The final survey contained 37 questions across ten content areas. The content areas and their purpose are presented in Table 2. All questions were closed-ended questions with mutually exclusive and exhaustive response options.

The final survey, informed consent form, advance letter, and cognitive interview guide are provided as Supplementary Materials.

## 4. Discussion

In this paper, we describe the development and dissemination of an HPV vaccination survey for American Indians in Cherokee Nation. As this survey is a new tool to advance our understanding of HPV vaccination factors in American Indian populations, we highlight our methodological choices and explain our decisions regarding the target population, sampling strategy, survey measures, and testing.

Increasing HPV vaccination rates in adolescents is an important step for cancer prevention efforts. It is a tribal priority affirmed in the Cherokee Nation Comprehensive Cancer Prevention and Control Plan 2018–2022 [17] and a national public health priority noted in the Healthy People 2030 initiative [47]. To advance and measure progress towards these priorities, there is a need for data that are specific to the tribe. Our survey was designed to estimate HPV vaccination coverage and assess factors associated with vaccination for American Indian adolescents aged 9 to 17 years in Cherokee Nation. By understanding vaccination factors, this survey will provide information to guide interventions and policies aimed towards improving HPV vaccine initiation and completion among Cherokee Nation adolescents (CONSIDER statement #17).

American Indian populations face data inequities. Existing data for their communities are often unavailable, inadequate, or incomplete [48,49]. In response to data inequities, Native nations are working towards advancing Indigenous data sovereignty and governance. Indigenous data sovereignty is “the right of a nation to govern the collection, ownership, and application of its own data” [50]. In a Tribal Data Practices Survey conducted by the National Congress of American Indians in 2017, responses representing 122 tribes revealed that 83% indicated that it was extremely important for tribes to collect or access data on their tribal citizens for governance purposes [49]. In the same survey, health (57%) was one of the most frequently identified need areas for more or better data on their tribal members [49]. Our survey represents a small effort among the numerous initiatives of data sovereignty and governance at Cherokee Nation. This project could provide guidance for other indigenous populations on how to conduct their research and evaluation projects to understand the health of their communities. Further, this project may guide academic and other research-based institutions on working with tribal entities and facilitate research and evaluation projects that are tribally directed and engaged. This may lead to better implementation of interventions aimed at improving the health and well-being of indigenous populations.

We anticipate several limitations with our survey. First, we plan to estimate HPV vaccination coverage for American Indian adolescents based on self-reported data provided by the parents. As a result, parental reports may under- or over-estimate the number of doses or shots received. However, previous studies have shown high concordance [51,52], indicating that parental reports of HPV vaccination status may be reasonably accurate. Second, and closely related, we rely on parental recall for HPV vaccination factors, which may vary depending on the time elapsed and information regarding the adolescents’ immunization, among other factors. Third, self-reported responses are subject to social desirability bias and may not correlate with actual or future behaviors, such as the intention to receive the HPV vaccine. However, we tried to minimize any resulting bias by assuring the participants in the consent letter that their responses were confidential. Fourth, we anticipate low response rates based on prior surveys of HPV vaccination [53,54], but have provided an incentive for participation, sent an advance letter, and mailed a reminder letter to mitigate nonresponse. In addition, we will adjust for nonresponse in our analysis. Fifth, due to the dynamic nature of the COVID-19 pandemic and response, participants’ attitudes and perceptions regarding the COVID-19 vaccine may evolve and not reflect the current reasons for vaccination. Lastly, we asked parents to select only the main reason for vaccinating or not vaccinating their child. Some parents may have had multiple reasons; however, capturing the main reasons allowed us to identify the most important reasons.

## 5. Conclusions

In summary, we developed a survey to estimate HPV vaccination coverage and factors. Survey design is a multi-step, collaborative, and iterative process. Through this paper, we have explained some of the steps with a view to make the process transparent and replicable. Researchers and communities interested in developing surveys for similar American Indian populations may benefit from the information presented in this paper.

## Figures and Tables

**Table 1:** Examples of select revisions to survey questions based on testing.

Content Area	Feedback	Rationale	Source	Final Revised Question
HPV awareness	Remove the following sentence from the question: HPV is different from HIV.	Participants felt that the sentence was unnecessary.	Cognitive interview	Human papillomavirus, also known as HPV, is a common sexually transmitted virus that can cause genital warts, cervical and other types of cancer in men and women. Before this survey, had you ever heard of human papillomavirus or HPV?
HPV and other vaccinations status	Remove the following sentence from the question: the Tdap booster shot also protects against pertussis or whooping cough.	Participants shared that the additional information on protection would be unnecessary.	Cognitive interview	Has your child ever received a tetanus booster shot? This is usually given at 11–12 years of age. The tetanus booster shot is also known as or sometimes called the Td or Tdap shot or vaccine.
HPV vaccine intent	Revise the question on intent (original question: how likely is it that your child will receive HPV shots in the next 12 months? Would you say)	Participants stated that the revised question would be much easier for parents or guardians to understand.	Cognitive interview	Will your child receive an HPV shot or vaccine in the next 12 months?
HPV vaccine recommendation	Include the following new question: Before this survey, did you know that an HPV shot or vaccine was recommended for children aged 9–17 years?	Participants stated that a question was needed to assess the awareness of the HPV vaccine recommendation.	Cognitive interview	Before this survey, did you know that an HPV shot or vaccine was recommended for children aged 9–17 years?
	Change “health care professional” to “health care provider”	Participants stated that the term “health care provider” would be more accurate.	Cognitive interview	Has a doctor, nurse, or other health care provider ever recommended that your child receive an HPV shot or vaccine?
HPV vaccine refusal	Remove the word “refuse” from the question.	Participants shared that the word “refuse” might be judgmental and alienate some participants.	AIDCoP meeting	If your child has NOT received any shots of the HPV vaccine, what is the MAIN reason that your child has NOT received the HPV shot or vaccine? (select ONE response only).
	Bold and capitalize the following words in the question: not and main	Participants stated that bolding and capitalizing the words would help with clarity and emphasis.	Cognitive interview	
HPV vaccine beliefs	Revise the following statement: The HPV vaccine is effective in preventing several cancers.	Participants asked to include and specify cervical cancer in the statement.	AIDCoP meeting	The HPV vaccine prevents cervical and other cancers.

Abbreviations: AIDCoP, American Indian Data Community of Practice; HPV, human papillomavirus.

**Table 2:** Information about the American Indian HPV Vaccination Survey in Cherokee Nation.

#	Content Area	# of Question(s)	Purpose
1	Eligibility	2	To assess participant eligibility for the survey.
2	Demographic information	11	To obtain demographic data from the survey participants.
3	HPV awareness	1	To assess awareness of the virus.
4	HPV vaccine awareness	1	To assess awareness of the HPV vaccine.
5	HPV and other vaccinations status	5	To assess uptake of the HPV, meningitis, and tetanus booster vaccines.
6	HPV vaccination intent	1	To assess intent to receive the HPV vaccine (if not already received).
7	HPV vaccine recommendation	4	To assess whether the HPV vaccine was discussed or recommended by a health care provider.
8	HPV vaccine refusal	1	To assess the main reason why the adolescent has not received the HPV vaccine.
9	HPV vaccine acceptance	1	To assess the main reason why the adolescent received the HPV vaccine.
10	HPV vaccine beliefs	7	To assess beliefs about the importance, safety, and side effects of the HPV vaccine.
11	COVID-19 and vaccination	3	To assess intent to receive the COVID-19 vaccine and whether the pandemic has caused difficulty in getting the HPV vaccine for the adolescent.

Abbreviations: HPV, human papillomavirus. # Total number of questions in each content area.

## Data Availability

The survey, informed consent form, advance letter, and cognitive interview guide are provided as Supplementary Materials.

## Supplementary Materials

The following are available online at https://www.mdpi.com/article/10.3390/ijerph18179239/s1, Supplementary File 1: Final Survey; Supplementary File 2: Informed Consent Form; Supplementary File 3: Advance Letter; Supplementary File 4: Cognitive Interview Guide.
