# Supplementary material for: Development and Implementation of an HPV Vaccination Survey for American Indians in Cherokee Nation
Source: Int J Environ Res Public Health. 2021 Sep 1;18(17):9239. doi: 10.3390/ijerph18179239 (PMC8431656; doi:10.3390/ijerph18179239)
Supplement: Supplementary file 1 [file ijerph-18-09239-s001.zip › Supplementary File 1_Final Survey.pdf]

American Indian HPV Vaccination Survey

Study ID Number: _ _ _ _

Instructions

Thank you for participating in this survey.

Please complete **<u>either</u>** the online or paper option, but not both.

You may complete this survey online at the following weblink:

https://redcap.link/hpvsurvey

OR

Complete this paper survey. For each question, please circle only one answer. There are no right or wrong answers.

After you complete the survey, kindly return the survey using the prepaid envelopes provided.

………

1. Eligibility

1.1. Do you currently have a child between 9-17 years of age living in your household?

1. Yes
2. No [Unfortunately, we are only surveying households with children between the ages of 9 to 17 years. Thank you for your time. You may stop taking the survey]

1.2. How many children do you have between the ages of 9 and 17 years?

1. 1
2. 2
3. 3
4. 4
5. 5 or more

………

Human Research Participant Protection The University of Oklahoma IRB APPROVED AAHRPP
IRB NUMBER: 12246
IRB APPROVAL DATE: 01/29/2021

If more than one child between the ages of 9- and 17-years lives with you, please think about the youngest when you answer the questions in this survey.

2. Demographic Information

Please answer questions about your youngest child in this section.

2.1. What is the gender of your child?
1. Male
2. Female
3. Other: _______________
4. Prefer not to answer

2.2. What is the race of your child? Circle all that apply
1. White
2. Black or African American
3. American Indian or Alaska Native
4. Asian
5. Native Hawaiian or Pacific Islander
6. Prefer not to answer

2.3. Is your child of Hispanic or Latino origin?
1. Yes
2. No
3. Don't know
4. Prefer not to answer

2.4. What is the age of your child?
1. 9 years
2. 10 years
3. 11 years
4. 12 years
5. 13 years
6. 14 years
7. 15 years
8. 16 years
9. 17 years
10. Prefer not to answer

As a parent or guardian, please answer questions about yourself in this section.

2.5. What is your gender?
1. Male
2. Female
3. Other: _______________
4. Prefer not to answer

2.6. What is your race? Circle all that apply
1. White
2. Black or African American
3. American Indian or Alaska Native
4. Asian
5. Native Hawaiian or Pacific Islander
6. Prefer not to answer

2.7. Are you of Hispanic or Latino origin?
1. Yes
2. No
3. Don't know
4. Prefer not to answer

2.8. What is your age in years?
1. _______________ years
2. Prefer not to answer

2.9. What is your relationship to the child? Are you the:
1. Mother (biological, step, foster, adoptive)
2. Father (biological, step, foster, adoptive)
3. Grandparent
4. Sister or brother (biological, step, foster, half, adoptive)
5. Aunt or uncle
6. Other: ________________
7. Prefer not to answer

2.10. What is your marital status?
1. Married or in a domestic partnership
2. Widowed
3. Divorced
4. Separated
5. Never married
6. Other: ________________
7. Prefer not to answer

2.11. What is your highest level of education?
1. Some high school
2. High school diploma or GED completed
3. Some college credit
4. Bachelor's degree
5. Master's, doctorate, or professional degree
6. Other: _______________
7. Prefer not to answer

IRB APPROVED
IRB NUMBER: 12246
IRB APPROVAL DATE: 01/29/2021

HPV Vaccine Questions

3.1. Human papillomavirus, also known as HPV, is a common sexually transmitted virus that can cause genital warts, cervical and other types of cancer in men and women.

Before this survey, had you ever heard of human papillomavirus or HPV?

1. Yes
2. No

………

4.1. A vaccine to prevent HPV infection is available. The vaccine is also known as or sometimes called the HPV vaccine, cervical cancer vaccine, Gardasil, or Cervarix.

Before this survey, had you ever heard of the HPV vaccine?

1. Yes
2. No

………

5.1. Has your child ever received an HPV shot or vaccine?

1. Yes
2. No
3. I don't know

5.2. How old was your child when they received their first HPV shot or vaccine?

1. Did not receive any HPV shot or vaccine
2. 9 years old or younger
3. 10 years old
4. 11 years old
5. 12 years old
6. 13 years old
7. 14 years old
8. 15 years old
9. 16 years old
10. 17 years old
11. I don't know

5.3. HPV vaccine is often given in multiple doses. How many shots of the HPV vaccine has your child ever received?

1. None
2. 1 shot
3. 2 shots
4. 3 or more shots
5. I don't know

IRB APPROVED
IRB NUMBER: 12246
IRB APPROVAL DATE: 01/29/2021

5.4. Has your child ever received a meningitis shot or vaccine, sometimes called Menactra, Menveo, or Menomune?

1. Yes
2. No
3. I don't know

5.5. Has your child ever received a tetanus booster shot? This is usually given at 11-12 years of age. The tetanus booster shot is also known as or sometimes called the Td or Tdap shot or vaccine.

1. Yes
2. No
3. I don't know

………

6.1. Will your child receive an HPV shot or vaccine in the next 12 months?

1. Yes
2. No
3. I don't know
4. My child has already received shot(s) of the HPV vaccine

………

7.1. Before this survey, did you know that an HPV shot or vaccine was recommended for children aged 9-17 years?

1. Yes
2. No

7.2. Has a doctor, nurse, or other health care provider ever recommended that your child receive an HPV shot or vaccine?

1. Yes
2. No
3. I don't know

7.3. At what age did the doctor, nurse, or other health care provider recommend that your child should start receiving the HPV shot or vaccine?

1. Before the age of 11 years
2. 11 to 14 years
3. 15 to 17 years
4. 18 years or older
5. No recommendation was ever made
6. I don't know

IRB APPROVED
IRB NUMBER: 12246
IRB APPROVAL DATE: 01/29/2021

7.4. Does your child typically receive their yearly well-child visits at a Cherokee Nation Health clinic or Hastings hospital?

1. Yes
2. No
3. I don't know

………

8.1. If your child has **NOT** received any shots of the HPV vaccine, what is the **<u>MAIN</u>** reason that your child has **NOT** received the HPV shot or vaccine? (select **ONE** response only)

1. My child has received the HPV vaccine.
2. I have not heard of the HPV vaccine.
3. I do not have enough information about the HPV vaccine
4. I do not have enough information about the diseases caused by HPV.
5. The vaccine was not recommended to my child by a doctor, nurse, or other health care provider.
6. The vaccine was not offered to my child by a doctor, nurse, or other health care provider.
7. The vaccine was not available in the provider's office.
8. My child was ill at the time of the visit.
9. My child's school does not require the vaccine.
10. I had concerns about the safety or the side effects of the vaccine.
11. I had concerns about the cost of the vaccine.
12. My child is not sexually active.
13. I had concerns that the vaccine may make my child sexually active.
14. I had difficulty making or getting to appointments with my child, such as transportation problems.
15. My child is afraid of needles.
16. Other. Please specify: ____

………

9.1. If your child has received any shots of the HPV vaccine, what is the **<u>MAIN</u>** reason that your child **DID** receive the HPV shot or vaccine? (select **ONE** response only)

1. My child has not received the HPV vaccine.
2. The vaccine was recommended to my child by a doctor, nurse, or other health care provider.
3. The vaccine was offered to my child by a doctor, nurse, or other health care provider.
4. I had enough information about the HPV vaccine.
5. I wanted to protect my child against HPV-associated cancer.
6. I wanted to protect my child against HPV infections.
7. I wanted to protect my child's future partner against HPV-associated cancer or HPV infections.
8. The vaccine was offered for free or at little cost.
9. My child has a high risk of getting an HPV infection.
10. Other. Please specify: ____

………

IRB APPROVED
IRB NUMBER: 12246
IRB APPROVAL DATE: 01/29/2021

HPV Vaccine Beliefs

How much do you agree or disagree with the following statements?

10.1 The HPV vaccine prevents cervical and other cancers.

1 = Strongly disagree
2 = Somewhat disagree
3 = Neither disagree or agree
4 = Somewhat agree
5 = Strongly agree

10.2 The HPV vaccine is important for my child’s health.

1 = Strongly disagree
2 = Somewhat disagree
3 = Neither disagree or agree
4 = Somewhat agree
5 = Strongly agree

10.3 In general, vaccines are important for my child’s health.

1 = Strongly disagree
2 = Somewhat disagree
3 = Neither disagree or agree
4 = Somewhat agree
5 = Strongly agree

10.4 The HPV vaccine can cause side effects, such as fever and discomfort.

1 = Strongly disagree
2 = Somewhat disagree
3 = Neither disagree or agree
4 = Somewhat agree
5 = Strongly agree

10.5 The HPV vaccine can cause lasting health problems.

1 = Strongly disagree
2 = Somewhat disagree
3 = Neither disagree or agree
4 = Somewhat agree
5 = Strongly agree

10.6 I have enough information about the HPV vaccine to decide whether or not to give it to my child.

1 = Strongly disagree
2 = Somewhat disagree
3 = Neither disagree or agree
4 = Somewhat agree
5 = Strongly agree

10.7 I am likely to get the HPV vaccine for my child in the next year.

1 = Strongly disagree
2 = Somewhat disagree
3 = Neither disagree or agree
4 = Somewhat agree
5 = Strongly agree
6 = Not applicable

……………………………………………………………………………………………………

IRB APPROVED
IRB NUMBER: 12246
IRB APPROVAL DATE: 01/29/2021

COVID-19 and Vaccination

11.1. How likely are <u>you</u> to get an approved COVID-19 vaccine when it becomes available?

1. I already got a COVID-19 vaccine
2. Very likely
3. Fairly likely
4. Not too likely
5. Not at all likely
6. Definitely not
7. Don't know
8. Prefer not to answer

11.2. How likely are you to get an approved COVID-19 vaccine for <u>your child</u> when it becomes available?

1. My child already got a COVID-19 vaccine
2. Very likely
3. Fairly likely
4. Not too likely
5. Not at all likely
6. Definitely not
7. Don't know
8. Prefer not to answer

11.3. Has the COVID-19 pandemic made it difficult to get the HPV vaccine for your child?

1. Yes
2. No
3. I don't know
4. My child has already received the HPV vaccine

………

You have reached the end of the survey.

We would like to thank you on behalf of the Cherokee Nation Health Services for the time and effort you have spent answering these questions. Again, thank you for investing your time to provide us feedback.

Kindly return the survey using the prepaid envelopes provided.

If you would like to receive a gift card for your participation, please complete the next page.

IRB APPROVED
IRB NUMBER: 12246
IRB APPROVAL DATE: 01/29/2021

Gift Card

Please complete the following information. This information will only be used for the purpose of providing a gift card.

After receiving the completed survey, we will send a gift card by mail to the following address.

Name ______________________________

Address ______________________________

____

Phone number ______________________________

Human Research Participant Protection
The University of Oklahoma
IRB APPROVED
AAHRPP
IRB NUMBER: 12246
IRB APPROVAL DATE: 01/29/2021
