# Supplementary material for: Development and Implementation of an HPV Vaccination Survey for American Indians in Cherokee Nation
Source: Int J Environ Res Public Health. 2021 Sep 1;18(17):9239. doi: 10.3390/ijerph18179239 (PMC8431656; doi:10.3390/ijerph18179239)
Supplement: Supplementary file 1 [file ijerph-18-09239-s001.zip › Supplementary File 2_Informed Consent Form.pdf]

American Indian HPV Vaccination Survey Informed Consent Statement

Please keep this document for your records.

Dear Prospective Participant:

Cherokee Nation is conducting a survey of the knowledge, vaccination status, and beliefs related to the human papillomavirus (HPV) vaccine. These surveys are being given to parents or guardians of American Indian children between the ages of 9 to 17 years who use Cherokee Nation Health Services. Your participation will help us increase HPV vaccination rates in Cherokee Nation. Your participation will also help to improve services and programs aimed at preventing or decreasing certain cancers within Cherokee Nation.

Procedure

We will survey about 2000 parents or guardians of American Indian children between the ages of 9 to 17 years. The survey will collect information on the health of 9 to 17-year-olds. We are only surveying households that have American Indian children between the ages of 9 to 17 years. The survey will take about 12-15 minutes to complete. The survey will include questions about HPV vaccine knowledge, status, and beliefs.

Confidentiality

You will not be identified with the information you give because the survey is confidential. No one but the survey team will know how you answered the questions on the survey. The survey team will keep all information about you confidential. The identifying information and surveys will be destroyed immediately after the data have been entered and analyzed. Only the survey team will have access to the study data. We will not use your name when we report the results of the survey. The information we collect from you will be combined with information from other participants to help develop a profile of Cherokee Nation surrounding HPV vaccination and vaccine-related behaviors.

Risks and Benefits

You may feel uncomfortable with some of the questions included in this survey. Some of the survey questions may be sensitive in nature. You do not have to answer any question you are not comfortable with and skip questions you do not want to answer. You can stop the survey at any time. The likely benefits to you are minimal; however, the overall impact for your tribal community will be important because new information on health behaviors will become available to address certain critical health problems. Further, you will receive a $10.00 gift card to Walmart for your participation.

Rights as a Volunteer

Your participation in this survey is voluntary. If you decide not to take part in or to stop the survey, you will not lose any services to which you are otherwise entitled. If for some

Human Research Participant Protection
The University of Oklahoma
IRB APPROVED
AAHRPP
IRB NUMBER: 12246
IRB APPROVAL DATE: 01/29/2021

reason you are unable to complete the survey, the completed information you provide, unless you tell us not to, will be combined with information from other participants to help develop a profile of community surrounding HPV vaccination and vaccine-related behaviors.

I have some names and phone numbers of people at Cherokee Nation that you can call if you have any questions about the survey. If you have any questions about this survey project, you may call Cherokee Nation epidemiologist, Dr. Ashley Comiford, at (918) 453-5000x7076. If you have questions about your rights, you may call Cherokee Nation Institutional Review Board Chair, Dr. Sohail Khan, at (918) 453-5602.

By taking the survey, you are agreeing to participate in this study.

Human Research Participant Protection
The University of Oklahoma
IRB APPROVED
AAHRPP
IRB NUMBER: 12246
IRB APPROVAL DATE: 01/29/2021
