# Supplementary material for: Development and Implementation of an HPV Vaccination Survey for American Indians in Cherokee Nation
Source: Int J Environ Res Public Health. 2021 Sep 1;18(17):9239. doi: 10.3390/ijerph18179239 (PMC8431656; doi:10.3390/ijerph18179239)
Supplement: Supplementary file 1 [file ijerph-18-09239-s001.zip › Supplementary File 3_Advance Letter.pdf]

ᏣᎳᎩᏯ ᎠᏰᎵ
CHEROKEE NATION®
P.O. Box 948 • Tahlequah, OK 74465-0948
918-453-5000 • www.cherokee.org

Office of the Chief

Chuck Hoskin Jr.
Principal Chief

Bryan Warner
Deputy Principal Chief

Hello Parent/Guardian of <INSERT NAME>,

I am Dr. Ashley Comiford, an epidemiologist, with Cherokee Nation Public Health. We are working to conduct a survey about human papillomavirus (HPV) vaccination, and we need your help.

The survey is called the Human Papillomavirus (HPV) Vaccination among American Indian Adolescents in Cherokee Nation. In the next few days, you will receive a survey in the mail. We ask that you take some time to answer our questions.

Please know that everything you tell us will be kept private. Your answers will help us to understand why parents/guardians decide or decline to get the HPV vaccine for their child. Your participation will also help to improve services and programs aimed at preventing or decreasing certain cancers within Cherokee Nation.

The survey will take approximately 15 minutes of your time. We hope that you will take part in the survey. Your participation is voluntary. You may choose not to participate or answer any question. You will not lose any benefits or receive any penalties if you choose not to participate in the survey.

Please contact me if you have any questions at 918-453-5000 ext. 7076 or at ashley-comiford@cherokee.org.

We are grateful for your help. Thank you for your cooperation.

Sincerely,

Ashley Comiford

Dr. Ashley Comiford, DrPH
Epidemiologist
Cherokee Nation Public Health

Human Research Participant Protection
The University of Oklahoma
IRB APPROVED
AAHRPP
IRB NUMBER: 12246
IRB APPROVAL DATE: 12/15/2020
