# Supplementary material for: Development and Implementation of an HPV Vaccination Survey for American Indians in Cherokee Nation
Source: Int J Environ Res Public Health. 2021 Sep 1;18(17):9239. doi: 10.3390/ijerph18179239 (PMC8431656; doi:10.3390/ijerph18179239)
Supplement: Supplementary file 1 [file ijerph-18-09239-s001.zip › Supplementary File 4_Cognitive Interview Guide.pdf]

Cognitive Interview Guide

Section I. Informed Consent

Before we start, I'm going to go over the consent form together.

Please feel free to ask any questions you might have as we go through the form.

- Cherokee Nation is conducting a survey of the knowledge, vaccination status, and beliefs related to the human papillomavirus (HPV) vaccine. We are developing surveys that will be given to parents or guardians of American Indian children between the ages of 9 to 17 years who use Cherokee Nation Health Services. The purpose of the interview is to help review some of the questions in the HPV Vaccination Survey to ensure the questions are well understood.

- The interview will take approximately 60 minutes and there are no right or wrong answers.

- You will receive $30 for your participation in the study.

- Your participation is voluntary and you may stop at any time.

- You may choose not to answer any questions you don't want to answer.

- If you have any questions about this survey project, you may call Cherokee Nation epidemiologist, Dr. Ashley Comiford, at (918) 453-5000x7076.

- If you have questions about your rights, you may call Cherokee Nation Institutional Review Board Chair, Dr. Sohail Khan, at (918) 453-5602.

Do you have any questions about the consent form?

☐ Yes

☐ No

Do you consent to the interview?

☐ Yes

☐ No

Human Research Participant Protection
The University of Oklahoma
IRB APPROVED
AAHRPP
IRB NUMBER: 12246
IRB APPROVAL DATE: 08/31/2020

Section II. Cognitive Interview

SAY

Now, if you are ready, I would like to get started asking you some of the questions from the HPV Vaccination Survey. This survey would be a mail or an online survey. It will be answered by a parent or guardian of a child between the age of 9 to 17 years. Therefore, if you could imagine yourself as a parent of an adolescent child when answering these questions. As I said earlier, this is NOT any kind of test and there are no right or wrong answers. We are reviewing these questions with American Indian people in Cherokee Nation to see how different people interpret the questions. Finally, after we are finished, you will receive a $30 gift card as an expression of our appreciation.

………

GENERAL PROBES (to be used as necessary):

- Please tell me what you are thinking.
- How did you choose your answer or how did you arrive at that answer?
- Could you tell me more about that?
- I'm not asking you to give me an answer, but could you tell me why you don't want to answer that question?
- Could you tell what you were thinking?

………

SAY

Let's begin.

3. HPV Awareness

3.1. Human papillomavirus also known as HPV is a common sexually transmitted virus that can cause genital warts, cervical and other types of cancer in men and women. HPV is different from HIV.

Before this survey, had you ever heard of human papillomavirus or HPV?

1. Yes

2. No

Human Research Participant Protection
The University of Oklahoma
IRB APPROVED
AAHRPP
IRB NUMBER: 12246
IRB APPROVAL DATE: 08/31/2020

According to you, what is this question asking about?

____

____

____

According to you, is this question asking about HPV or HIV?

____

____

____

According to you, was there any confusion about the question?

____

____

____

……………………………………………………………………………………………………

4. HPV Vaccine Awareness

4.1. A vaccine to prevent HPV infection is available. The vaccine is called HPV vaccine, cervical cancer vaccine, Gardasil, or Cervarix.

Before this survey, had you ever heard of the HPV vaccine?

1. Yes
2. No

Can you tell me in your own words what this question is asking?

____

____

____

Human Research Participant Protection
The University of Oklahoma
IRB APPROVED
AAHRPP
IRB NUMBER: 12246
IRB APPROVAL DATE: 08/31/2020

You said ______________________. Can you tell me more please?

According to you, were the brand names, Gardasil or Cervarix, helpful?

5. HPV and Other Vaccinations Status

5.1. Has your child ever received an HPV shot or vaccine?

1. Yes ➔ [Go to question **5.2**]
2. No ➔ [Go to question **5.4**]
3. I don't know

Can you tell me in your own words what this question is asking?

Did you have any difficulty answering this question?

Human Research Participant Protection
The University of Oklahoma
IRB APPROVED
IRB NUMBER: 12246
IRB APPROVAL DATE: 08/31/2020

5.2. How many shots of the HPV vaccine has your child ever received?

1. None
2. 1 shot
3. 2 shots
4. 3 or more shots
5. I don't know

Did you have any difficulty answering this question?

____

____

____

Is there another way you might ask this question?

____

____

____

……

5.3. How old was your child when they received their first HPV shot?

1. Did not receive any HPV shots
2. 9 years old or younger
3. 10 years old
4. 11 years old
5. 12 years old
6. 13 years old
7. 14 years old
8. 15 years old
9. 16 years old
10. 17 years old

Human Research Participant Protection
The University of Oklahoma
IRB APPROVED
AAHRPP
IRB NUMBER: 12246
IRB APPROVAL DATE: 08/31/2020

11. I don't know

Can you tell me in your own words what this question is asking?

____

____

____

According to you, was there any confusion in answering this question?

____

____

____

……

5.4. Has your child ever received a meningitis shot or vaccine, sometimes called Menactra, Menveo, or Menomune?

1. Yes
2. No
3. I don't know

What do you think this question is asking?

____

____

____

Did you have any difficulty answering this question?

____

____

____

Human Research Participant Protection
The University of Oklahoma
IRB APPROVED
AAHRPP
IRB NUMBER: 12246
IRB APPROVAL DATE: 08/31/2020

Is there another way you might ask this question?

5.5. Has your child ever received a tetanus booster shot, such as Td or Tdap? The Tdap booster shot also protects against pertussis or whooping cough.

1. Yes
2. No
3. I don't know

What do you think this question is asking?

What does the word "booster shot" mean to you?

Was there any confusion in answering this question?

Human Research Participant Protection
The University of Oklahoma
IRB APPROVED
AAHRPP
IRB NUMBER: 12246
IRB APPROVAL DATE: 08/31/2020

6. HPV Vaccination Intent

6.1. How likely is it that your child will receive HPV shots in the next 12 months? Would you say:

1. Very Likely
2. Somewhat Likely
3. Not very likely
4. Not at all likely
5. My child has already received shot(s) of the HPV vaccine

What do you think this question is asking?

Did you have any difficulty answering this question?

Is there another way you might ask this question?

....

7. HPV Vaccination Recommendation

7.1. Has a doctor or other health care professional ever recommended that your child receive HPV shots?

1. Yes
2. No

Human Research Participant Protection
The University of Oklahoma
IRB APPROVED
AAHRPP
IRB NUMBER: 12246
IRB APPROVAL DATE: 08/31/2020

3. I don't know

What do you think this question is about?

Did you have any difficulty answering this question?

………

7.2. At what age did the doctor or health care professional recommend that your child should start receiving the HPV shots?

1. Before age 11
2. 11 or 12 years
3. 13 or 14 years
4. 15 or 16 years
5. 17 or 18 years
6. After 18 years
7. No specific age was recommended or discussed
8. I don't know

What is this question asking?

Human Research Participant Protection
The University of Oklahoma
IRB APPROVED
AAHRPP
IRB NUMBER: 12246
IRB APPROVAL DATE: 08/31/2020

Is there another way you might ask this question?

___

___

___

………

7.3. Does your child typically receive their well-child visits at Cherokee Nation Health Services?

1. Yes
2. No
3. I don't know

What do you think this question is asking?

___

___

___

What does the word "well-child visits" mean to you?

___

___

___

Is there another way you might ask this question?

___

___

___

………

Human Research Participant Protection
The University of Oklahoma
IRB APPROVED
AAHRPP
IRB NUMBER: 12246
IRB APPROVAL DATE: 08/31/2020

8. HPV Vaccine Refusal

8.1. If your child has not received any or all shots of the HPV vaccine, what is the MAIN reason that your child has NOT received the HPV vaccine?

1. My child has received the HPV vaccine.
2. I have not heard of the HPV vaccine.
3. I do not have much information about the HPV vaccine
4. I do not have much information about the diseases caused by HPV.
5. The vaccine was not recommended by a doctor or other health care provider.
6. The vaccine was not offered to my child by a doctor or other health care provider.
7. The vaccine was not available in the provider's office.
8. My child was ill at the time of the visit.
9. My child's school does not require the vaccine.
10. I had concerns about the safety of the vaccine.
11. I had concerns about the cost of the vaccine.
12. My child is not sexually active.
13. I had concerns that the vaccine may make my child sexually active.
14. I had difficulty making or getting to appointments with my child, such as transportation problems.
15. My child is afraid of needles.
16. Other. Please specify: ______________________________________

In your own words, what do you think this question is asking?

____

____

____

Did you have any difficulty answering this question?

____

____

____

Human Research Participant Protection
The University of Oklahoma
IRB APPROVED
AAHRPP
IRB NUMBER: 12246
IRB APPROVAL DATE: 08/31/2020

Was there any confusion about answering this question?

____

____

____

For each response option, could you tell me if you have any difficulty with the response option or if there is another way to phrase the response option?

**Response options**	**Do you have any difficulty with the response option?**	**Is there another way to phrase the response?**
1. My child has received the HPV vaccine.		
2. I have not heard of the HPV vaccine.		
3. I do not have much information about the HPV vaccine		
4. I do not have much information about the diseases caused by HPV.		
5. The vaccine was not recommended by a doctor or other health care provider.		
6. The vaccine was not offered to my child by a doctor or other health care provider.		
7. The vaccine was not available in the provider's office.		
8. My child was ill at the time of the visit.		
9. My child's school does not require the vaccine.		
10. I had concerns about the safety of the vaccine.		
11. I had concerns about the cost of the vaccine.		
12. My child is not sexually active.		

Human Research Participant Protection
The University of Oklahoma
IRB APPROVED
IRB NUMBER: 12246
IRB APPROVAL DATE: 08/31/2020

13. I had concerns that the vaccine may make my child sexually active.		
14. I had difficulty making or getting to appointments with my child, such as transportation problems.		
15. My child is afraid of needles.		

………

9. HPV Vaccine Acceptance

9.1. If your child has received any or all shots of the HPV vaccine, what is the MAIN reason that your child DID receive the HPV vaccine?

1. My child has not received the HPV vaccine.
2. The vaccine was recommended by a doctor or other health care provider.
3. The vaccine was offered to my child by a doctor or other health care provider.
4. I had knowledge about the vaccine.
5. I wanted to protect my child against cancer.
6. I wanted to protect my child against HPV infections.
7. I wanted to protect my child's future partner.
8. The vaccine was offered for free or at little cost.
9. My child has a high probability of contracting or acquiring HPV infection.
10. Other. Please specify: ______________________________________

In your own words, what do you think this question is asking?

____

____

____

Did you have any difficulty answering this question?

____

Human Research Participant Protection
The University of Oklahoma
IRB APPROVED
AAHRPP
IRB NUMBER: 12246
IRB APPROVAL DATE: 08/31/2020

____

____

Was there any confusion about answering this question?

____

____

____

**Response options**	**Do you have any difficulty with the response option?**	**Is there another way to phrase the response?**
1. My child has not received the HPV vaccine.		
2. The vaccine was recommended by a doctor or other health care provider.		
3. The vaccine was offered to my child by a doctor or other health care provider.		
4. I had knowledge about the vaccine.		
5. I wanted to protect my child against cancer.		
6. I wanted to protect my child against HPV infections.		
7. I wanted to protect my child's future partner.		
8. The vaccine was offered for free or at little cost.		
9. My child has a high probability of contracting or acquiring HPV infection.		

....

SAY

Human Research Participant Protection
The University of Oklahoma
IRB APPROVED
AAHRPP
IRB NUMBER: 12246
IRB APPROVAL DATE: 08/31/2020

I am going to read you some statements, could you say if you have any difficulty or confusion with the statement or is there another way to phrase the statement. All the statement will have a response option ranging from “strongly disagree”, “somewhat disagree”, “neither disagree or agree”, “somewhat agree”, and “strongly agree”

**Statements**	**Do you have any difficulty or confusion with the response option?**	**Is there another way to phrase the response?**
1. The HPV vaccine is effective in preventing cervical and other cancers.		
2. The HPV vaccine is important for my child’s health.		
3. Vaccines other than the HPV vaccine are important for my child’s health.		
4. The HPV vaccine causes short-term health problems, such as fever and discomfort.		
5. The HPV vaccine causes lasting health problems.		
6. I don’t have enough information about the HPV vaccine to decide whether to give it to my child.		
7. I am likely to get the HPV vaccine for my child in the next year.		

IRB NUMBER: 12246
IRB APPROVAL DATE: 08/31/2020
